# Complete chloroplast genome sequence of *Sophora japonica* ‘JinhuaiJ2’ (Papilionaceae), an important traditional chinese herb

**DOI:** 10.1080/23802359.2019.1703588

**Published:** 2019-12-18

**Authors:** Yancai Shi, Bingbing Liu

**Affiliations:** a Guangxi Institute of Botany, Guangxi Zhuang Autonomous Region, Chinese Academy of Sciences, Guilin, China;; b Institute of Loess Plateau, Shanxi University, Taiyuan, China

**Keywords:** Papilionaceae, Chloroplast genome, Phylogenetic analysis

## Abstract

*Sophora japonica* ‘JinhuaiJ2’ (Papilionaceae), known as Chinese Scholar Tree, is an important traditional herb with a long history of cultivation in China. It’s well known for its valuable medicinal values due to its flower buds contains abundant rutin. Here, we reported and characterized its complete chloroplast genome based on Illumina paired-end sequencing data. The complete plastid genome was 158,690 bp, which contained inverted repeats (IR) of 25,339 bp separated by a large single-copy (LSC) and a small single copy (SSC) of 88,978 bp and 19,034 bp, respectively. The cpDNA contains 129 genes, comprising 83 protein-coding genes, 38 tRNA genes and 8 rRNA genes. The overall GC content of the plastome is 36.1%. The phylogenetic analysis of 18 selected chloroplast genomes demonstrated that *S. japonica* ‘JinhuaiJ2’ was close to the species *Tapiscia sinensis*.

*Sophora japonica* ‘JinhuaiJ2’ (Papilionaceae), which belongs to the Leguminosae family, is an important traditional Chinese herb with a long history of cultivation (Sun et al. 2007). Its flower buds contain abundant flavonoids which are used as a hemostatic agent in traditional Chinese medicine, and therefore, widely used in industrial extraction of an active pharmaceutical ingredient, rutin, which is frequently reported to exert positive effects in animal body metabolism, including anti-platelet, antioxidant, and anti-inflammatory (Kim and Yun-Choi 2008). Herein, we reported and characterized its complete plastome based on Illumina paired-end sequencing data, which will contribute to the further studies on its genetic research and resource utilization. The annotated cp genome of *S. japonica* ‘JinhuaiJ2’ has been deposited into GenBank with the accession number MN701078.

In this study, *S. japonica* ‘JinhuaiJ2’ was sampled from in Guangxi Zhuang Autonomous Region of China, located at 110°18′33″ E, 25°3′37″ N. A voucher specimen (Shi Y.-C. et al. H1316) was deposited in the Guangxi Key Laboratory of Plant Conservation and Restoration Ecology in Karst Terrain, Guangxi Institute of Botany, Guangxi Zhuang Autonomous Region and Chinese Academy of Sciences, Guilin, China. The experiment procedure is as reported in Zhang et al. (2019). Around 2 Gb clean data were used for the cp genome de novo assembly by the program NOVOPlasty (Dierckxsens et al. 2017) and direct-viewing in Geneious R11 (Biomatters Ltd., Auckland, New Zealand). Annotation was performed with the program Plann (Huang and Cronk 2015) and Sequin (http://www.ncbi.nlm.nih.gov/.).

The chloroplast genome of *S. japonica* ‘JinhuaiJ2’ is a typical quadripartite structure with a length of 158,690 bp, which contained inverted repeats (IR) of 25,339 bp separated by a large single-copy (LSC) and a small single copy (SSC) of 88,978 bp and 19,034 bp, respectively. The cpDNA contains 129 genes, comprising 83 protein-coding genes, 38 tRNA genes and 8 rRNA genes. Among the annotated genes, 16 of them contain one intron (*atp*F, *ndh*A, *ndh*B, *rps*12, *rps*16, *rpoC*1, *pet*B, *pet*D, *rpl*16, *rpl*2, *trn*A-UGC, *trn*I-GAU, *trn*K-UUU, *trn*L-UAA, *trn*T-CGU and *trn*V-UAC), and two genes (*clp*P and *ycf*3) contain two introns. The overall GC content of the plastome is 36.1%.

To identify the phylogenetic position of *S. japonica* ‘JinhuaiJ2’, phylogenetic analysis was conducted. A neighbor joining (NJ) tree with 1000 bootstrap replicates was inferred using MEGA version 7 (Kumar et al. 2016) from alignments created by the MAFFT (Katoh and Standley 2013) using plastid genomes of 18 species. It showed the position of *S. japonica* ‘JinhuaiJ2’ was close to the species *Tapiscia sinensis* (Figure 1). Our findings can be further used for plastome evolution and phylogenomic studies of Papilionaceae. It will also provide fundamental data for the utilization and management of this important medicinal plant.

**Figure 1.**
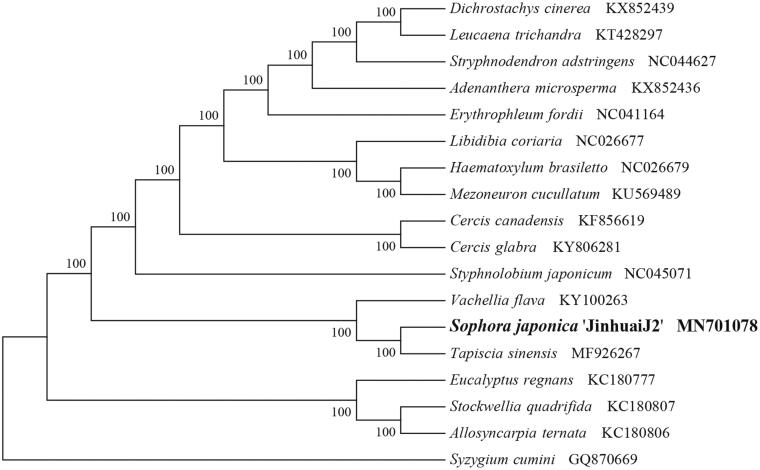
NJ phylogenetic tree of Sophora *japonica* ‘JinhuaiJ2’ with 18 species was constructed by chloroplast plastome sequences. Numbers on the nodes are bootstrap values from 1000 replicates. *Syzygium cumini* was selected as outgroups.
